# Intensity-specific leisure-time physical activity and depressive symptoms among first-year university students: a four-wave longitudinal study

**DOI:** 10.7717/peerj.21498

**Published:** 2026-07-02

**Authors:** Xinyang Lu, Lianghao Zhu, Lunxin Chen, Miaomiao Wen, Lingzi Yao

**Affiliations:** 1 School of Physical Education and Sports, Central China Normal University, Wuhan, Hubei, China; 2 Key Laboratory of Psychological and Physiological Regulation in Competitive Sports, Tianjin University of Sport, Tianjin, China; 3 School of Physical Education, Guangzhou Maritime University, Guangzhou, Guangdong, China

**Keywords:** Public health, Physical activity, Latent growth model, Mental health

## Abstract

**Background:**

Depressive symptoms are common among first-year university students, yet the longitudinal associations between different intensities of leisure-time physical activity (LTPA) and mental health remain unclear. This study examined the relationships between moderate- and vigorous-intensity LTPA and depressive symptoms over one academic year, as well as potential gender differences.

**Methods:**

A four-wave longitudinal survey was conducted over one academic year among 456 freshmen (Mage = 18.18, SD = 0.67; 202 males and 254 females). Latent growth modeling was employed to analyze temporal changes in depressive symptoms and LTPA, and a parallel process latent growth model was used to assess the relationships between LTPA intensity and depressive symptom trajectories.

**Results:**

Depressive symptoms were relatively elevated at the beginning of the academic year and showed a modest decline over time, whereas LTPA increased across waves. The intercepts of vigorous-intensity LTPA and depressive symptoms were significantly and negatively associated (*β* =  − 0.441, *p* = 0.002), whereas the association between moderate-intensity LTPA and depressive symptoms at baseline was not statistically significant (*β* =  − 0.278, *p* = 0.071). Importantly, no significant associations were observed between changes in LTPA and changes in depressive symptoms over time (moderate: *β* =  − 0.187, *p* = 0.508; vigorous: *β* =  − 0.061, *p* = 0.823). Female students reported lower baseline levels of both moderate- and vigorous-intensity LTPA compared with males; however, no significant gender differences were observed in the longitudinal associations between LTPA and depressive symptoms.

**Conclusions:**

Vigorous-intensity LTPA and depressive symptoms were negatively associated at baseline among first-year university students. However, changes in LTPA were not significantly related to changes in depressive symptoms over the study period. These findings underscore the importance of distinguishing between baseline associations and longitudinal change when interpreting relationships between physical activity and mental health.

## Introduction

The early stage of university life represents a critical transitional period that is often accompanied by multiple challenges. During this time, students must adapt to new academic, social, and personal environments while managing academic pressure, psychological and physiological changes, lifestyle adjustments, and the reorganization of interpersonal and family relationships (Huang et al., 2025; Li et al., 2022; Sheldon et al., 2021). Without effective coping mechanisms, students are particularly vulnerable to elevated levels of depressive symptoms. Globally, the prevalence of depression among university students remains alarmingly high. A large-scale meta-analysis reported a pooled prevalence of 33.6%, with the highest rates observed in Africa (40.1%), followed by Asia (34.8%) and North America (35.5%) (Mokdad et al., 2016). Among young adults, depression is a major contributor to years lived with disability and disability-adjusted life years (Mokdad et al., 2016) and is strongly associated with premature mortality and suicidal behaviors (Patel et al., 2016; Walker, McGee & Druss, 2015). Consequently, identifying effective strategies to mitigate depressive symptoms has become an urgent public health priority.

Physical activity (PA), as a positive health behavior, has been widely recognized as an effective means of reducing the risk of depressive symptoms (Rodriguez-Ayllon et al., 2019; Schuch et al., 2016; Schuch et al., 2018). Compared with traditional psychotherapeutic or pharmacological interventions, PA offers distinct advantages—such as accessibility, low cost, and minimal side effects—making it particularly suitable for university populations. Mounting evidence suggests that the beneficial effects of PA on mental health are driven by both psychosocial and neurophysiological mechanisms (Lubans et al., 2016; Singh et al., 2023). From a psychosocial perspective, engaging in diverse physical activities may enhance self-regulation and alleviate stress (Lubans et al., 2016). From a neurophysiological standpoint, PA improves depressive symptoms through multiple biological pathways, including the upregulation of neurotrophic factors, increased availability of serotonin and norepinephrine, and reduced systemic inflammation (Gujral et al., 2017; Lubans et al., 2016). Moreover, participation in PA enhances adolescents’ physical self-perception, self-worth, and self-esteem (Lubans et al., 2016), all of which contribute to better emotional well-being.

A systematic review encompassing 49 prospective cohort studies with 266,939 participants demonstrated a significant association between higher levels of PA and a reduced risk of depression across age groups and geographical regions (Schuch et al., 2018). Similarly, a meta-analysis of 25 randomized controlled trials (RCTs) found that PA exerted a significant antidepressant effect compared with control conditions (Schuch et al., 2016). In addition, a comprehensive review and meta-analysis of 114 original studies—including both intervention and observational designs—revealed that PA interventions significantly improved adolescents’ mental health and that PA was inversely associated with depressive symptoms (Rodriguez-Ayllon et al., 2019). Recent studies focusing on university students further support the negative correlation between physical activity and depressive symptoms (Zhang et al., 2024; Zhao, Deng & Zhou, 2025; Zhao et al., 2024).

In the present study, we focus specifically on leisure-time physical activity (LTPA) rather than total PA. First, domain-specific evidence suggests that the relationship between physical activity and mental health differs across activity domains, with leisure-time activity showing (Patel et al., 2016; Walker, McGee & Druss, 2015) more consistent beneficial associations with mental health than some non-leisure domains of PA (White et al., 2017). Second, compared with total PA, LTPA is more discretionary and more strongly shaped by individual choice, motivation, and campus opportunities, making it particularly relevant during the transition into university life. Finally, from a practical perspective, university-based physical activity promotion typically targets voluntary and recreational forms of activity rather than occupational, household, or transport-related activity, making LTPA a more relevant and actionable behavioral domain for first-year students (Donnelly, Penny & Kynn, 2024; Huang et al., 2024).

Although substantial evidence suggests that physical activity (PA) is associated with a lower risk of depression, the dose or level of PA required to achieve this benefit remains unclear. Paolucci et al. (2018) found that moderate-intensity PA reduced depression, anxiety, and perceived stress by lowering proinflammatory cytokines, whereas vigorous-intensity PA—despite its antidepressant potential—might increase perceived stress and inflammation due to heightened physiological strain. In contrast, VanKim & Nelson (2013) reported that vigorous-intensity PA was associated with better mental health and lower perceived stress among university students, potentially through enhanced social interactions. Interestingly, Li et al. (2024) observed that both moderate- and vigorous-intensity PA were strongly correlated with lower depression risk. These inconsistent findings highlight the need for more nuanced research to clarify how PA intensity relates to depressive symptoms.

Gender is another factor related to both LTPA participation and depressive symptoms during the first-year transition. However, evidence is mixed regarding whether PA—depression associations differ by gender. For example, one study suggested that males derive greater benefits from PA, as they tend to engage more actively in physical activities that facilitate emotional regulation (Liu et al., 2024). Whereas other work indicates that regular PA may provide stronger benefits for females experiencing mild to moderate depression (Zhang & Yen, 2015). Given these inconsistencies—and because gender differences in activity participation and stress exposure may be salient during early university life—we examine gender-related patterns exploratorily in this study.

Although previous research has provided some evidence for an association between physical activity and depressive symptoms (Zhang et al., 2024; Zhao, Deng & Zhou, 2025; Zhao et al., 2024), the longitudinal relationship between intensity-specific leisure-time physical activity (LTPA) and depressive symptoms remains unclear, particularly among first-year university students undergoing a critical developmental transition. A longitudinal approach is needed to distinguish between two conceptually distinct yet equally important questions: first, whether differences in students’ activity levels at university entry are associated with differences in depressive symptoms; and second, whether changes in activity levels over time are accompanied by changes in depressive symptoms. This gap is important at both theoretical and practical levels. Theoretically, it limits understanding of whether the LTPA—depression relationship primarily reflects stable baseline differences or developmental co-change over time. Practically, it makes it difficult for universities to determine whether mental health promotion should focus more on identifying high-risk students with low initial activity levels or on promoting sustained behavioral change in activity across the academic year. To address this gap, the present study employed a four-wave longitudinal design and applied a parallel process latent growth model (PP-LGM), which simultaneously estimates the intercepts (initial levels) and slopes (rates of change) of depressive symptoms and intensity-specific LTPA. This approach allows us to examine both baseline associations (intercept–intercept) and the coupling of developmental change (slope–slope). The primary contribution of the present study is to distinguish baseline associations from coupled developmental change in intensity-specific LTPA and depressive symptoms during the first year of university.

This study addressed two central questions: (1) What are the initial levels and developmental trajectories of depressive symptoms and LTPA among first-year university students? and (2) Are baseline levels of overall, vigorous-intensity, and moderate-intensity LTPA associated with baseline depressive symptoms, and are changes in LTPA coupled with changes in depressive symptoms over time? Based on prior evidence (Rodriguez-Ayllon et al., 2019; Schuch et al., 2016; Schuch et al., 2018), we expected higher overall LTPA to be associated with lower initial levels of depressive symptoms. Given the inconsistent findings regarding PA intensity (Li et al., 2024; Paolucci et al., 2018; VanKim & Nelson, 2013), intensity-specific associations were examined in an exploratory manner rather than with strong directional hypotheses. In addition, given the mixed evidence on gender-related differences in PA and mental health (Liu et al., 2024; Zhang & Yen, 2015), we explored gender-related differences in trajectories and associations by including gender as a time-invariant covariate.

## Methods

### Participants and procedures

Portions of this text were previously published as part of a preprint (https://doi.org/10.21203/rs.3.rs-8409125/v1). Participants were first-year undergraduate students from a comprehensive university in Central China, representing a range of academic disciplines including education, literature, engineering, management, and the arts. Ethical approval for this study was obtained from the Ethics Committee (Institutional Review Board, IRB) of Central China Normal University (approval no. CCNU-IRB-202509002A). Informed consent was obtained electronically from all participants prior to participation. Before completing the online questionnaire, participants were provided with detailed information about the purpose of the study, the voluntary nature of participation, and assurances of confidentiality and anonymity. Only participants who indicated their consent by selecting the consent option proceeded to the survey. All questionnaires were completed online during scheduled sessions and submitted immediately upon completion.

This study adopted a one academic year longitudinal research design with four waves across two semesters, and data were collected at three-month intervals to fully capture temporal changes. Specifically, data for T1 and T2 were collected in the first week and the last week of the first semester, respectively, while data for T3 and T4 were collected in the first week and the last week of the second semester, respectively. The time spacing between each wave was designed to balance sensitivity to developmental change with the need to minimize respondent burden. The procedure was identical across all measurement occasions, and each assessment took approximately 15 min to complete.

A longitudinal cohort of 456 first-year students was identified for follow-up across four waves during the first academic year (mean age = 18.18 years; 202 male and 254 female students). The figure of 456 refers to the full set of unique participants in the longitudinal cohort, whereas the wave-specific counts refer to valid questionnaire responses for the study variables at each wave. At T1, 381 valid questionnaires were obtained. The corresponding numbers of valid responses at T2, T3, and T4 were 426, 390, and 452, respectively. Because all 456 students were invited to participate at each wave, students who did not provide valid data at an earlier wave were allowed to re-enter the study at later waves. This re-entry structure explains why the number of valid responses at T4 exceeded that at T1. Across the four waves, the wave-specific responses summed to 1,649 valid questionnaire responses, corresponding to 1,649 valid person-wave observations. At the participant level, 281 students provided valid data at all four waves, whereas the remaining 175 students provided valid data at three waves; no participant provided valid data at only one or two waves. Thus, all participants included in the analytic sample contributed data from at least three waves. The analytic sample for PP-LGM consisted of all 456 unique participants. Missing data were handled using full-information maximum likelihood, allowing all available observations to contribute to parameter estimation under the missing-at-random assumption.

To assess the pattern of missing data, Little’s MCAR test was conducted, yielding nonsignificant results (*χ*^2^ = 26.33, *df* = 24, *p* > 0.05), indicating that the data were missing completely at random (MCAR). In accordance with recommendations by Duncan, Duncan & Li (1998) and Chen, Savalei & Rhemtulla (2020), missing data were handled using maximum likelihood estimation with robust standard errors (MLR) in subsequent analyses. Nevertheless, if nonparticipation at particular waves was related to unmeasured psychological or contextual factors, some bias may still remain; therefore, the findings should be interpreted with appropriate caution.

### Measures

#### Depressive symptoms

Depressive symptoms were assessed using the Chinese version of the Patient Health Questionnaire-9 (PHQ-9; Kroenke, Spitzer & Williams, 2001), a widely used self-report instrument designed to evaluate the presence and severity of depressive symptoms. The PHQ-9 consists of nine items, each corresponding to the core diagnostic criteria for major depressive disorder outlined in the Diagnostic and Statistical Manual of Mental Disorders, Fourth Edition (DSM-IV). Participants were instructed to respond to the question: “During the past two weeks, how often have you been bothered by the following problems in your daily life?” Responses were recorded on a 4-point Likert-type scale ranging from 0 (not at all) to 3 (nearly every day). Total scores range from 0 to 27, calculated as the sum of all item scores, with higher scores indicating more severe depressive symptoms. Following the classification proposed by Paolucci et al. (2018), depressive symptom severity was categorized into five levels: minimal (0–4), mild (5–9), moderate (10–14), moderately severe (15–19), and severe (20–27).

The PHQ-9 has demonstrated robust psychometric properties across diverse populations, and the Simplified Chinese version has been validated among both adolescents and adults, exhibiting excellent reliability and validity (Leung et al., 2020; Tsai et al., 2014). In the present study, the PHQ-9 showed high internal consistency across all four waves (*α* = 0.880 at T1; *α* = 0.908 at T2; *α* = 0.921 at T3; *α* = 0.938 at T4).

#### Leisure-Time Physical Activity (LTPA)

Leisure-time physical activity (LTPA) was measured using the Godin–Shephard Leisure-Time Physical Activity Questionnaire (GSLTPAQ; Godin & Shephard, 1997), a widely adopted instrument for assessing habitual physical activity in free time. Participants were asked to report, during a typical 7-day period, how many times they engaged in vigorous, moderate, and light exercise for more than 15 min during their free time. In accordance with the original GSLTPAQ instructions, intensity categories were defined by the questionnaire’s exertion descriptors and example activities, rather than by researcher-defined MET thresholds or later recoding. Specifically, vigorous-intensity LTPA was described as activity that makes the heart beat rapidly (*e.g.*, running, soccer, basketball), moderate-intensity LTPA as activity that is not exhausting (*e.g.*, fast walking, volleyball, easy swimming), and light-intensity LTPA as activity involving minimal effort (*e.g.*, yoga, golf, easy walking).

Thus, participants classified their own activities according to these standardized descriptors and examples. For scoring, the weekly frequencies of vigorous, moderate, and light activity were weighted by nine, five, and three, respectively, and summed to yield the total LTPA score; these values were used for score calculation rather than for defining the intensity categories. The GSLTPAQ has demonstrated good reliability and validity when compared with objective measures such as accelerometer data and maximal oxygen uptake (VO_2_max) (Amireault & Godin, 2015). In the present study, the LTPA scale showed acceptable internal consistency across all four waves (*α* = 0.714 at T1; *α* = 0.778 at T2; *α* = 0.796 at T3; *α* = 0.762 at T4).

### Statistical analysis

Statistical analyses were conducted in several sequential steps. First, SPSS 26.0 was used to perform descriptive statistics, repeated-measures analyses of variance (ANOVA), and correlation analyses to examine the distributional characteristics of all variables and their interrelationships. Next, Mplus 8.3 was employed to construct unconditional latent growth models (LGM) for depressive symptoms, overall leisure-time physical activity (LTPA), and different intensities of LTPA, in order to explore their developmental trajectories over time. In this framework, the intercept factor represents the initial level of the construct, while the slope factor reflects its rate of change over time. Subsequently, PP-LGM were estimated to evaluate the associations between the growth components (*i.e.,* intercepts and slopes) of depressive symptoms and LTPA. In the PP-LGM framework, the previously established univariate LGM were incorporated into a single integrated model to assess the dynamic relationships among the latent intercepts and slopes of all variables, while gender was included as a covariate. Specifically, gender was entered as a time-invariant covariate predicting the intercepts and slopes of depressive symptoms and LTPA. Model fit was evaluated using multiple goodness-of-fit indices, including the chi-square to degrees of freedom ratio (*χ*^2^/df), the Comparative Fit Index (CFI), the Tucker–Lewis Index (TLI), the Root Mean Square Error of Approximation (RMSEA), and the Standardized Root Mean Square Residual (SRMR). Model fit was considered acceptable when CFI and TLI values exceeded 0.90, and good when both were greater than 0.95. Values of RMSEA below 0.08 and SRMR below 0.06 were also indicative of a good model fit (Hu & Bentler, 1999).

## Results

### Descriptive statistics and correlation analysis

A repeated-measures ANOVA was first conducted to examine the main effects of time, gender, and their interaction on the study variables. For depressive symptoms, Mauchly’s test of sphericity indicated a violation of the sphericity assumption (*W* = 0.938, *p* < 0.05); therefore, the Greenhouse–Geisser correction was applied. The results revealed a significant main effect of time (*F* = 6.953, *p* < 0.001), while neither the main effect of gender (*F* = 1.348, *p* > 0.05) nor the interaction effect between time and gender (*F* = 1.542, *p* > 0.05) reached significance. Similarly, for LTPA, Mauchly’s test also indicated that the assumption of sphericity was violated (*W* = 0.819, *p* < 0.001), and thus the Greenhouse–Geisser correction was used. The results showed a significant main effect of time (*F* = 25.432, *p* < 0.001) and a significant main effect of gender (*F* = 57.419, *p* < .001), but the interaction effect between time and gender was not significant (*F* = 0.659, *p* > 0.05).

As shown in Table 1, the mean scores for depressive symptoms (DS) fluctuated across the four time points, with standard deviations ranging from 4.71 to 4.90, suggesting a slight increase in interindividual variability. The mean score of overall LTPA showed a continuous upward trend, while its standard deviation first decreased and then increased, indicating that students’ overall participation in leisure-time physical activity improved over time, with intra-group differences narrowing initially and slightly widening later. Both vigorous- and moderate-intensity LTPA displayed similar trends—mean levels increased steadily, and standard deviations decreased before rising again—suggesting that participation in both activity intensities increased, with temporal fluctuations in within-group variability.

**Table 1:** Descriptive statistics for depressive symptoms and different intensities of leisure-time physical activity. Descriptive statistics of depressive symptoms and the intensity of physical activity (LTPA) in different leisure time points in four points.

		**Time1**	**Time2**	**Time3**	**Time4**
	**Range**	**M/SD**	**M/SD**	**M/SD**	**M/SD**
**DS**	0–27	5.89/4.71	6.28/4.62	5.45/4.69	5.60/4.90
**Overall** ** LTPA**	0–119	42.57/24.69	50.15/20.02	50.45/20.59	55.76/23.44
**V-LTPA**	0–63	20.53/15.44	25.56/12.78	25.64/13.18	28.10/14.66
**M-LTPA**	0–35	12.87/8.69	14.91/6.35	15.21/6.60	16.13/8.00

**Notes.**

Overall LTPA Overall Leisure-Time Physical Activity V-LTPA vigorous-intensity Leisure-Time Physical Activity M-LTPA moderate intensity Leisure-Time Physical Activity DS depression symptoms

Table 2 presents the correlations between depressive symptoms (DS) and overall LTPA, vigorous-intensity LTPA, and moderate-intensity LTPA across the four measurement occasions. Overall, depressive symptoms were negatively correlated with different intensities of LTPA at most time points. Specifically, overall LTPA and vigorous-intensity LTPA exhibited consistent and significant negative correlations with depressive symptoms across most waves, whereas correlations with moderate-intensity LTPA were weaker or nonsignificant, particularly at T2 and T4. These findings suggest that higher levels of physical activity—especially vigorous-intensity LTPA—are generally associated with lower depressive symptoms, although the strength of this association appears to vary across time and activity intensity, warranting further longitudinal verification.

**Table 2:** Correlations between depressive symptoms and different intensities of leisure-time physical activity. Correlations between depressive symptoms and overall LTPA, vigorous-intensity LTPA, and moderate-intensity LTPA across the four measurement occasions.

	**1**	**2**	**3**	**4**	**5**	**6**	**7**	**8**	**9**	**10**	**11**	**12**	**13**	**14**	**15**	**16**
**DS**_**T1**_	1															
**DS**_**T2**_	0.438^**^	1														
**DS**_**T3**_	0.393^**^	0.555^**^	1													
**DS**_**T4**_	0.357^**^	0.501^**^	0.576^**^	1												
**Overall LTPA**_**T1**_	−0.134^**^	−0.173^**^	−0.122^*^	−0.161^**^	1											
**Overall LTPA**_**T2**_	−0.130^*^	−0.119^*^	−0.091	−0.096^*^	0.296^**^	1										
**Overall LTPA**_**T3**_	−0.170^**^	−0.127^*^	−0.174^**^	−0.158^**^	0.162^**^	0.392^**^	1									
**Overall LTPA**_**T4**_	−0.061	−0.052	−0.059	−0.106^*^	0.131^*^	0.223^**^	0.284^**^	1								
**V-LTPA**_**T1**_	−0.145^**^	−0.156^**^	−0.153^**^	−0.182^**^	0.895^**^	0.255^**^	0.180^**^	0.134^**^	1							
**V-LTPA**_**T2**_	−0.140^**^	−0.169^**^	−0.122^*^	−0.075	0.288^**^	0.925^**^	0.377^**^	0.244^**^	0.266^**^	1						
**V-LTPA**_**T3**_	−0.167^**^	−0.128^*^	−0.168^**^	−0.140^**^	0.169^**^	0.397^**^	0.925^**^	0.269^**^	0.197^**^	0.411^**^	1					
**V-LTPA**_**T4**_	−0.112^*^	−0.056	−0.081	−0.108^*^	0.157^**^	0.223^**^	0.271^**^	0.911^**^	0.171^**^	0.251^**^	0.290^**^	1				
**M-LTPA**_**T1**_	−0.095	−0.131^*^	−0.062	−0.136^**^	0.813^**^	0.216^**^	0.085	0.152^**^	0.536^**^	0.193^**^	0.069	0.151^**^	1			
**M-LTPA**_**T2**_	−0.044	0.003	0.027	−0.067	0.200^**^	0.805^**^	0.284^**^	0.125^*^	0.152^**^	0.571^**^	0.246^**^	0.095	0.161^**^	1		
**M-LTPA**_**T3**_	−0.116^*^	−0.058	−0.111^*^	−0.131^*^	0.07	0.249^**^	0.807^**^	0.235^**^	0.079	0.189^**^	0.562^**^	0.182^**^	0.066	0.259^**^	1	
**M-LTPA**_**T4**_	0.034	−0.026	−0.016	−0.057	0.071	0.115^*^	0.182^**^	0.825^**^	0.081	0.116^*^	0.143^**^	0.571^**^	0.098	0.101^*^	0.195^**^	1

**Notes.**

Overall LTPA Overall Leisure-Time Physical Activity V-LTPA_TI−T4_ vigorous-intensity Leisure-Time Physical Activity Time1-4 M-LTPA_T1−T4_ moderate-intensity Leisure-Time Physical Activity Time1-4 DS_TI−T4_ depression symptoms Time 1-4

** *p* < 0.01.

* *p* < 0.05.

### Measurement invariance

Table 3 presents the results of the measurement invariance tests conducted for the depression scale, to ensure that the observed changes over time reflect true developmental changes rather than variations in the measurement structure across time points (Liao et al., 2022). In this study, a series of increasingly restrictive models were estimated to assess measurement invariance across the four time points, including configural invariance (equal factor structure), weak invariance (equal factor loadings), and strong invariance (equal item intercepts). Given that the chi-square (*χ*^2^) statistic is highly sensitive to sample size, changes in fit indices (ΔCFI, ΔTLI, and ΔRMSEA) were used to evaluate invariance between the configural and more constrained models. Measurement invariance was considered to be established when ΔCFI and ΔTLI were both less than 0.01, and ΔRMSEA was less than 0.015.

**Table 3:** Fit statistics for measurement invariance testing of depressive symptoms. Results of the measurement invariance tests conducted for the depression scale, to ensure that the observed changes over time reflect true developmental changes rather than variations in the measurement structure across time points.

**Model**	*χ* ^2^	***df***	**CFI**	**ΔCFI**	**TLI**	**ΔTLI**	**RMSEA**	**ΔRMSEA**
Configural	1,148.381^*^	534	0.943	–	0.932	–	0.042	–
Weak	1,200.231^*^	558	0.941	−0.002	0.934	0.002	0.042	0
Strong	1,255.100^*^	582	0.937	−0.004	0.932	−0.002	0.042	0

**Notes.**

CFI Comparative Fit Index TLI Tucker–Lewis Index RMSEA Root Mean Square Error of Approximation

The results supported scalar invariance across the four waves. Scalar invariance indicates that, in addition to maintaining the same factor structure and factor loadings across time points, the item intercepts were equivalent over time. This level of invariance suggests that meaningful comparisons can be made regarding the latent mean levels of depressive symptoms across the four waves.

### Unconditional growth model

In this study, separate univariate unconditional growth models were constructed for depressive symptoms, overall LTPA, vigorous-intensity LTPA, and moderate-intensity LTPA. Overall model fit information and parameter estimate for the intercept and slope components are presented in Table 4. First, an unconditional linear growth model was established for depressive symptoms. The model demonstrated a good fit to the data (*χ*^2^/*df* = 2.515, CFI = 0.977, TLI = 0.972, RMSEA = 0.045, SRMR = 0.033). According to the parameter estimates, the initial level of depressive symptoms was 1.949 (SE = 0.195, *p* < 0.001), indicating that, on average, freshmen exhibited a mild level of depressive symptoms at the beginning of the first semester, with significant inter-individual variability. The mean slope was −0.183 (SE = 0.101, *p* > 0.05), suggesting a slight decreasing trend in depressive symptoms over time. The correlation between the intercept and slope was not significant (*r* = −0.096, *p* > 0.05), implying that the initial level of depressive symptoms was not associated with the rate of change.

**Table 4:** Overall model fit, level, and slope trajectories for the growth models. In this study, separate univariate unconditional growth models were constructed for depressive symptoms, overall LTPA, vigorous-intensity LTPA, and moderate-intensity LTPA. Overall model fit information and parameter estimates for the intercept and slope components are presented in this Table.

**Estimates of parameters**	**Depression**	**Overall leisure-time physical activity**	**Vigorous-intensity LTPA**	**Moderate-intensity LTPA**
**Means**				
**Intercept**	1.949^*^(0.195)	2.923^*^(0.000)	2.478^*^(0.000)	3.718^*^(0.000)
**Slope**	−0.183 (0.101)	0.920^*^(0.000)	1.173 (0.102)	0.864^*^(0.028)
**Correlation**				
**r**	−0.096 (0.680)	−0.610^*^(0.000)	−0.447^*^(0.207)	−0.563^*^(0.025)
**Fit of the model**				
	*χ*^2^/*df* = 2.515	*χ*^2^/*df* = 2.090	*χ*^2^/*df* = 1.592	*χ*^2^/*df* = 1.667
	*p* = 0.0845	*p* = 0.0948	*p* = 0.2426	*p* = 0.2298
	CFI = 0.977	CFI = 0.962	CFI = 0.985	CFI = 0.948
	TLI = 0.972	TLI = 0.955	TLI = 0.981	TLI = 0.938
	RMSEA = 0.045	RMSEA = 0.044	RMSEA = 0.027	RMSEA = 0.029
	90% CI [0–0.088]	90% CI [0–0.087]	90% CI [0–0.075]	90% CI [0–0.076]

**Notes.**

Standard errors are in parentheses.

** *p* < 0.01.

* *p* < 0.05.

Next, unconditional linear and quadratic growth models were fitted for overall LTPA, but both showed unsatisfactory model fit. Subsequently, a freely estimated latent basis model was tested by fixing the slope loadings for the first two time points to 0 and 1, and allowing the last two loadings to be freely estimated. The resulting model fit indices (*χ*^2^/*df* = 3.461, CFI = 0.941, TLI = 0.882, RMSEA = 0.071, SRMR = 0.039) indicated suboptimal fit as TLI was below 0.90. To improve model fit, a constrained latent basis model was estimated, fixing the slope loadings at 0, 1, 1.2, and 1.8 for the four time points. This model demonstrated good fit (*χ*^2^/*df* = 2.090, CFI = 0.962, TLI = 0.955, RMSEA = 0.044, SRMR = 0.039) and was therefore retained as the final model.

Results showed that the mean initial level of overall LTPA was 2.923 (SE = 0.386, *p* < 0.001), and the mean slope was 0.920 (SE = 0.249, *p* < 0.001), indicating that students’ engagement in leisure-time physical activity increased significantly across the academic year. The non-linear pattern of slope factor loadings suggests that the rate of increase was not constant over time. The correlation between the intercept and slope was significant and negative (*r* = −0.610, *p* < 0.001), implying that students with lower initial levels of LTPA tended to show greater increases over time.

### Unconditional growth model for moderate-intensity LTPA

Unconditional linear and quadratic growth models were first constructed for moderate-intensity LTPA; however, both models yielded unsatisfactory fit indices. Subsequently, a freely estimated latent basis model was tested, fixing the slope loadings for the first two time points to 0 and 1, while allowing the last two loadings to be freely estimated. The model fit indices (*χ*^2^/*df* = 2.329, CFI = 0.924, TLI = 0.847, RMSEA = 0.045, SRMR = 0.034) indicated that the model fit remained suboptimal, as TLI was below the recommended cutoff of 0.90. Based on the freely estimated loadings (0, 1, 1.223, and 1.583), a constrained latent basis model was then specified, fixing the slope loadings at 0, 1, 1.5, and 2 across the four measurement points. The revised model demonstrated a good overall fit (*χ*^2^/*df* = 1.677, CFI = 0.948, TLI = 0.938, RMSEA = 0.029, SRMR = 0.036) and was therefore retained as the final model.

Parameter estimates revealed that the mean initial level of moderate-intensity LTPA among freshmen was 3.718 (SE = 0.887, *p* < 0.001), while the mean slope was 0.864 (SE = 0.394, *p* < 0.05), indicating a significant upward trajectory over the academic year. The non-linear pattern of the slope loadings suggests that the rate of increase in moderate-intensity LTPA was not constant, implying accelerated engagement in such activities during later stages of the year. Moreover, the correlation between the intercept and slope was significantly negative (*r* = −0.563, *p* < 0.05), suggesting that students who initially engaged less in moderate-intensity LTPA exhibited greater increases in participation over time.

### Unconditional growth model for vigorous-intensity LTPA

Unconditional linear and quadratic growth models were first constructed for vigorous-intensity LTPA; however, both models exhibited unsatisfactory fit indices. To improve model performance, a freely estimated latent basis model was then tested, with slope loadings for the first two time points fixed at 0 and 1, and the remaining two loadings freely estimated. The resulting model fit indices (*χ*^2^/*df* = 2.626, CFI = 0.967, TLI = 0.935, RMSEA = 0.051, SRMR = 0.037) indicated that, although fit was acceptable overall, the 90% confidence interval (CI) of RMSEA exceeded 0.10, suggesting that further refinement was warranted. Based on the freely estimated slope loadings (0, 1, 1.058, and 1.485), a constrained latent basis model was subsequently specified by fixing the four slope loadings to 0, 1, 1.1, and 1.5, respectively. This revised model achieved a strong overall fit (*χ*^2^/*df* = 1.592, CFI = 0.985, TLI = 0.981, RMSEA = 0.027, SRMR = 0.038) and was therefore retained as the final model for vigorous-intensity LTPA.

Model estimates revealed that the mean initial level of vigorous-intensity LTPA among first-year students was 2.478 (SE = 0.567, *p* < 0.001), indicating relatively low engagement at the beginning of the academic year. The mean slope was 1.173 (SE = 0.717, *p* > 0.05), suggesting a non-significant upward trend in participation over time. Consistent with the pattern of the slope loadings, the rate of change in vigorous-intensity LTPA was non-linear, implying that increases in participation did not occur at a uniform pace. The correlation between intercept and slope was negative but non-significant (*r* = −0.447, *p* > 0.05), indicating that students’ initial levels of vigorous-intensity activity were not systematically related to the rate of change in their subsequent trajectories.

### Parallel process latent growth model

The parallel process latent growth models indicated that baseline levels (intercepts) of vigorous-intensity LTPA and depressive symptoms were significantly and negatively associated, whereas the corresponding baseline association for moderate-intensity LTPA was not statistically significant. In contrast, the slope–slope associations were consistently nonsignificant across models (overall, moderate-, and vigorous-intensity LTPA), suggesting no evidence that changes in LTPA were significantly coupled with changes in depressive symptoms over the academic year.

### PP-LGM of depression and overall LTPA

Next, the unconditional latent growth models for depression and overall LTPA were integrated to construct an unconditional parallel process latent growth model aiming to further examine the associations between the two constructs over time. The PP-LGM demonstrated an excellent overall fit (*χ*^2^/*df* = 1.382, CFI = 0.989, TLI = 0.987, RMSEA = 0.021, 90% CI [0.000–0.047], SRMR = 0.034). The covariances between the intercepts and slopes of the two variables revealed several key patterns. Specifically, the initial level of depression was significantly and negatively associated with the initial level of overall LTPA (*β* = −0.388, *p* = 0.001), suggesting that students with higher baseline depressive symptoms tended to engage in lower levels of LTPA. However, the initial level of depression was not significantly associated with the rate of change in LTPA (*β* = 0.219, *p* = 0.196), indicating that baseline depression did not substantially influence the trajectory of LTPA over time. Likewise, the initial level of LTPA was not significantly associated with change in depressive symptoms over time (*β* = 0.031, *p* = 0.828). The covariance between the slopes of depression and LTPA was also nonsignificant (*β* = −0.140, *p* = 0.512), implying that changes in the two constructs were not synchronized across time.

### Incorporating gender as a time-invariant covariate

Building upon the baseline PP-LGM, gender was included as a time-invariant covariate to examine whether it was associated with differences in the intercepts and slopes of the study variables. The results indicated that gender was not significantly associated with either the initial level (*β* = 0.059, *p* = 0.391) or the rate of change (*β* = 0.101, *p* = 0.299) of depression. However, gender had a significant negative effect on the initial level of LTPA (*β* = −0.385, *p* < 0.001), indicating that female students reported substantially lower initial levels of LTPA than their male counterparts. Gender was not significantly associated with the rate of change in LTPA (*β* = 0.033, *p* = 0.750). Furthermore, the initial levels of depression and LTPA were found to be significantly negatively correlated (*r* = −0.391, *p* = 0.001), suggesting that students with higher baseline depressive symptoms tended to participate less in leisure-time physical activity at the outset.

### PP-LGM of depression and moderate-intensity LTPA

Next, the unconditional latent growth models for depression and moderate-intensity LTPA were integrated to construct an unconditional parallel process latent growth model, aiming to further explore the longitudinal associations between these two variables. The model demonstrated a good overall fit (*χ*^2^/*df* = 1.489, CFI = 0.985, TLI = 0.981, RMSEA = 0.023, 90% CI [0.000–0.047], SRMR = 0.039). The covariance results between the intercepts and slopes of the two constructs revealed that the initial level of depression was negatively but not significantly associated with the initial level of moderate-intensity LTPA (*β* = −0.278, *p* = 0.071). The initial level of depression was not significantly associated with the rate of change in moderate-intensity LTPA (*β* = 0.269, *p* = 0.257), indicating that baseline depressive symptoms did not exert a substantial influence on the trajectory of moderate-intensity physical activity over time. Similarly, the initial level of moderate-intensity LTPA was not significantly associated with change in depressive symptoms over time (*β* = −0.073, *p* = 0.715), and the covariance between the slopes of depression and moderate-intensity LTPA was also nonsignificant (*β* = −0.187, *p* = 0.508), suggesting that changes in the two constructs were not dynamically coupled.

When gender was added as a time-invariant covariate, the results indicated that gender was not significantly associated with either the initial level (*β* = 0.060, *p* = 0.382) or the rate of change (*β* = 0.099, *p* = 0.309) of depression. However, gender was significantly associated with the initial level of moderate-intensity LTPA (*β* = −0.385, *p* = 0.001), indicating that female students exhibited significantly lower initial levels of moderate-intensity LTPA compared with male students. Gender was not significantly associated the rate of change in moderate-intensity LTPA (*β* = 0.058, *p* = 0.681). Additionally, the initial levels of depression and moderate-intensity LTPA were negatively correlated but not statistically significant (*r* = −0.274, *p* = 0.100). Similarly, the slopes of depression and moderate-intensity LTPA were not significantly correlated (*r* = −0.187, *p* = 0.484), indicating that the longitudinal changes in the two constructs were largely independent over time.

### PP-LGM of depression and vigorous-intensity LTPA

Next, the unconditional latent growth models for depression and vigorous-intensity LTPA were integrated to construct an unconditional parallel process latent growth model, with the goal of further examining the longitudinal association between depression and high-intensity leisure-time physical activity. The overall model demonstrated an excellent fit (*χ*^2^/*df* = 1.378, CFI = 0.990, TLI = 0.987, RMSEA = 0.021, 90% CI [0.000–0.047], SRMR = 0.033).

The covariance estimates between the intercepts and slopes indicated that the initial level of depression was significantly and negatively associated with the initial level of vigorous-intensity LTPA (*β* = −0.441, *p* = 0.002), suggesting that students with higher baseline depressive symptoms tended to engage in less vigorous-intensity physical activity at the beginning of the study. However, the initial level of depression was not significantly associated with the rate of change in vigorous-intensity LTPA over time (*β* = −0.198, *p* = 0.373), indicating that baseline depression did not substantially influence the rate at which vigorous-intensity physical activity changed. Similarly, the initial level of vigorous-intensity LTPA was not significantly associated with change in depressive symptoms over time (*β* = −0.020, *p* = 0.895) and the covariance between the slopes of depression and vigorous-intensity LTPA was also nonsignificant (*β* = −0.061, *p* = 0.823).

After including gender as a time-invariant covariate, the results showed that gender was not significantly associated with either the initial level (*β* = 0.058, *p* = 0.400) or the rate of change (*β* = 0.104, *p* = 0.289) of depression. However, gender was significantly associated with the initial level of vigorous-intensity LTPA (*β* = −0.471, *p* < 0.001), indicating that female students engaged in significantly less vigorous-intensity physical activity at baseline compared with male students. Gender was not significantly associated with the rate of change in vigorous-intensity LTPA (*β* = 0.150, *p* = 0.270). Moreover, after controlling for gender, the initial level of depression remained significantly and negatively correlated with the initial level of vigorous-intensity LTPA (*r* = −0.453, *p* = 0.004), suggesting that individuals with higher initial depressive symptoms tended to exhibit lower levels of vigorous-intensity LTPA participation at the outset. Table 5 presents the correlation coefficients between depressive symptoms and different intensities of leisure-time physical activity in the parallel process latent growth model.

**Table 5:** Correlation coefficients between depressive symptoms and different intensities of leisure-time physical activity in the parallel process latent growth model.

	**Overall LTPA intercept**	**Overall LTPA slope**	**V-LTPA intercept**	**V-LTPA slope**	**M-LTPA intercept**	**M-LTPA slope**
**DS intercept**	−0.388^**^	0.219	−0.441^**^	0.198	−0.332^*^	0.359
**DS slope**	0.031	−0.140	0.020	0.061	−0.070	−0.197

**Notes.**

Overall LTPA Overall Leisure-Time Physical Activity V-LTPA vigorous-intensity Leisure-Time Physical Activity M-LTPA moderate intensity Leisure-Time Physical Activity DS depression symptoms

** *p* < 0.01.

* *p* < 0.05.

## Discussion

The present study employed latent growth modeling to examine trajectories of depressive symptoms and leisure-time physical activity among first-year college students across one academic year, and to assess intensity-specific associations as well as gender differences by including gender as a time-invariant covariate. The findings revealed that first-year students exhibited a relatively high initial level of depression, which showed a marginally significant but slight downward trend over time. Moreover, the developmental trajectory of depressive symptoms demonstrated a nonlinear pattern, indicating that the rate of change in depression was not constant throughout the academic year. This finding is consistent with Sheldon et al. (2021), who noted that the transition to university represents a critical period of heightened vulnerability to depression. During this stage, students often face multiple, interrelated challenges—ranging from personal factors (*e.g.*, low self-confidence, poor self-esteem) and lifestyle issues (*e.g.*, unhealthy habits, poor sleep quality) to environmental stressors (*e.g.*, academic pressure, financial difficulties) and insufficient social support (*e.g.*, limited family or peer support) (Xu et al., 2021). These factors jointly contribute to emotional instability and increase the likelihood of depressive fluctuations among university freshmen. Taken together, these results underscore that first-year students constitute a population experiencing elevated depressive symptoms during the university transition. Importantly, the significant variance observed in both the initial levels and rates of change of depressive symptoms suggests substantial individual differences in students’ emotional adaptation. The absence of a significant correlation between the intercept and slope further implies that baseline symptom severity was not associated with the rate of change over time. From a practical perspective, these findings suggest that early identification and continued monitoring of student mental health during the first academic year may be valuable.

The present study further examined the patterns of leisure-time physical activity (LTPA) among first-year university students. The results revealed that students generally reported low levels of LTPA at the beginning of college, but their engagement increased steadily over the course of the academic year. This trend suggests that students gradually adapt to university life and develop stronger motivation and capacity to participate in physical activity as time progresses. One of the most prominent barriers to LTPA among university students is a perceived lack of time (Brown et al., 2024). During the initial phase of university transition, first-year students often prioritize academic achievement above all else, believing that academic success requires intensive effort and long study hours. Consequently, exercise is frequently the first activity sacrificed when time pressures arise (Hilger-Kolb, Loerbroks & Diehl, 2020). In addition, freshmen often struggle with ineffective time management and lifestyle adjustment during their early adaptation period, making it difficult to allocate consistent time for physical activity (Kwan & Faulkner, 2011). These factors collectively explain the low baseline level of LTPA observed at the start of the academic year. As the semester progresses, however, students appear to develop better coping strategies and integrate physical activity into their routines, resulting in a gradual increase in LTPA levels. This upward trajectory aligns with previous research showing that students tend to enhance both their motivation and self-efficacy for leisure-time exercise as they adapt to academic and social demands (Scarapicchia et al., 2017a; Scarapicchia et al., 2017b). A noteworthy finding was the negative association between the initial level of LTPA and its subsequent rate of change. Specifically, students with lower initial LTPA showed greater subsequent increases, whereas those with higher initial LTPA showed smaller subsequent increases. Future studies could examine whether differentiated or tiered approaches based on initial LTPA levels are feasible and effective for promoting sustained physical activity engagement.

Although the parallel process models indicated a significant association at baseline—such that overall and vigorous-intensity LTPA were related to depressive symptoms at initial status—no statistically significant slope–slope associations were observed for any LTPA intensity. This suggests that there was no evidence of coupled change between LTPA and depressive symptoms across the academic year. Similar null change-to-change findings have been reported in some longitudinal studies of leisure-time physical activity and depressive symptoms (Birkeland, Torsheim & Wold, 2009; Rothon et al., 2010; Toseeb et al., 2014). Importantly, this pattern is theoretically meaningful: baseline (intercept–intercept) associations primarily reflect between-person differences, whereas slope–slope associations test within-person co-development, and these two levels of association need not align. Several factors may help explain the absence of slope–slope coupling in the present study. First, depressive symptoms during the first year of university may be strongly shaped by time-varying contextual stressors (*e.g.*, adjustment challenges at semester entry and examination-related pressure toward semester end). Such stressors may influence both changes in activity and changes in mood, thereby obscuring any underlying co-change at the group level (Rothon et al., 2010). Second, the spacing of assessments (beginning *vs* end of semesters) may be too coarse to capture shorter-term dynamics between physical activity and affect that may operate on weekly or daily timescales. Finally, limited variability in individual change and measurement error inherent in self-reported physical activity may further reduce statistical power to detect slope–slope coupling (Birkeland, Torsheim & Wold, 2009).

The parallel process latent growth model examining depressive symptoms and overall leisure-time physical activity (LTPA) indicated a significant negative association between their initial levels (intercepts). Specifically, first-year students who reported higher depressive symptoms at college entry tended to report lower levels of LTPA at baseline. This pattern is consistent with prior evidence of an inverse association between physical activity and depressive symptoms (Rodriguez-Ayllon et al., 2019; Schuch et al., 2016; Schuch et al., 2018) and may reflect that students with higher depressive symptomatology at entry experience lower motivation, reduced energy, or fewer opportunities to engage in leisure-time physical activity during the early transition to university.

Importantly, the longitudinal coupling between developmental changes was not supported. Neither baseline LTPA significantly predicted subsequent change in depressive symptoms, nor did baseline depressive symptoms significantly predict change in LTPA, and the slope–slope association between LTPA and depressive symptoms was nonsignificant. Taken together, these results suggest that the observed relationship primarily reflects baseline between-person differences rather than synchronized within-person change over the academic year. Therefore, the present findings should not be interpreted as evidence that increasing LTPA leads to reductions in depressive symptoms over time.

A key contribution of the present study lies in examining vigorous- and moderate-intensity LTPA separately in relation to depressive symptoms using parallel process latent growth models. The findings indicated a significant negative association between the initial level of depressive symptoms and the initial level of vigorous-intensity LTPA (*β* = −0.441, *p* = 0.002), whereas the corresponding association for moderate-intensity LTPA was negative but not statistically significant (*β* = −0.278, *p* = 0.071). Furthermore, after adjusting for gender, the baseline association between vigorous-intensity LTPA and depressive symptoms remained statistically significant, suggesting that the intensity-specific pattern was more evident for vigorous-intensity LTPA than for moderate-intensity LTPA in this sample. However, because the study is observational and the slope–slope associations were nonsignificant, these findings should be interpreted as baseline associations rather than evidence of protective or intervention effects over time. This pattern contrasts with some earlier studies (Huang et al., 2025; Paolucci et al., 2018) reporting comparable associations across intensity levels.

The observed negative baseline association between vigorous-intensity LTPA and depressive symptoms may be understood in relation to both biological and psychosocial mechanisms (Saucedo Marquez et al., 2015; Schuch et al., 2016). From a biological perspective, several mechanisms may help explain why students with higher baseline vigorous-intensity LTPA tended to report lower baseline depressive symptoms. Vigorous-intensity physical activity may be associated with BDNF-related neuroplastic processes, which may be relevant to emotion-regulation regions such as the hippocampus and prefrontal cortex (Saucedo Marquez et al., 2015). It may also be associated with more favorable neural activation and inflammatory regulation, which could partially account for the baseline differences in depressive symptoms observed among students with different levels of vigorous-intensity LTPA (Andrews et al., 2020; Kandola et al., 2019).

From a psychosocial perspective, the stronger link between vigorous intensity LTPA and reduced depression may stem from its enhanced social and self-perceptual benefits. Engaging in vigorous activities often involves team-based or group settings (*e.g.*, basketball, soccer, collective training), where participants experience greater social interaction and peer support compared to moderate intensity activities (Li et al., 2022; Schuch et al., 2016). Such interactions foster a sense of belonging and companionship, which can alleviate loneliness and buffer against negative affect, thereby indirectly improving mental health outcomes (VanKim & Nelson, 2013). Moreover, vigorous intensity LTPA tends to enhance self-efficacy, body image, and self-esteem, which in turn promote better emotional regulation and resilience (Lubans et al., 2016). Together, these psychosocial processes may help explain why a stronger negative baseline association was observed for vigorous-intensity LTPA than for moderate-intensity LTPA in the present sample.

Another central aim of this study was to examine whether gender was associated with differences in the associations between LTPA and depressive symptom trajectories. In the PP-LGM, gender was included as a time-invariant covariate rather than being tested *via* formal moderation. The results showed that gender was not significantly associated with the intercept or slope of depressive symptoms, nor with the slopes of LTPA, suggesting no evidence of gender differences in developmental trajectories or in the longitudinal associations between LTPA and depressive symptoms over the academic year. This pattern contrasts with prior mixed evidence suggesting that men may derive greater mental health benefits from physical activity (Liu et al., 2024) or, conversely, that women may benefit more (Zhang & Yen, 2015). In addition, after adjusting for gender, the negative baseline association between depressive symptoms and LTPA remained evident, indicating that the association observed at initial status was not driven by gender.

Although no gender differences were observed in the longitudinal associations, gender differences were apparent in baseline LTPA levels. Specifically, females reported lower initial levels of total, vigorous-, and moderate-intensity LTPA than males, with the largest gap observed for vigorous-intensity activities. This pattern aligns with previous studies (Sisay, 2021; Xu et al., 2021) showing that female students tend to participate less frequently in high-intensity or competitive physical activities. Given the observational nature of the present study and the nonsignificant slope–slope associations, these findings should be interpreted as evidence of baseline disparities rather than direct support for specific intervention effects. Future research could examine the mechanisms underlying gender differences in baseline LTPA (*e.g.*, perceived barriers, self-efficacy) and test, using intervention or quasi-experimental designs, whether tailored strategies (*e.g.*, low-barrier, inclusive, and skill-development programs) can effectively reduce gender gaps in LTPA and influence mental health outcomes.

## Limitations

Several limitations should be considered when interpreting the present findings. First, both depressive symptoms and leisure-time physical activity were assessed using self-report measures, which may be affected by recall bias, reporting bias, and measurement error. In addition, the four waves were conducted at the beginning and end of two semesters, and students’emotional states during these periods may have been systematically influenced by time-varying contextual stressors, such as adaptation challenges at university entry, social relationship stress, academic demands, and examination pressure later in the semester. Consequently, some of the observed fluctuations in depressive symptoms may reflect semester-specific stress rather than changes associated with LTPA.

Second, several potentially important confounding variables were not measured or included in the analyses, including sleep patterns, academic stress, social support, prior mental health history, major life events, and family-related factors such as socioeconomic background, parental support, parenting style, or parental mental health. These unmeasured variables may influence both LTPA and depressive symptoms, and therefore residual confounding cannot be ruled out. In particular, information on pre-existing mental health conditions at baseline (*e.g.*, prior diagnosis or treatment) was not collected, so their potential influence could not be examined.

Third, although the four-wave design allowed us to distinguish baseline associations from developmental change, the findings were more consistent with baseline associations than with longitudinal coupling between changes in LTPA and changes in depressive symptoms. The nonsignificant slope–slope associations indicate limited evidence that changes in LTPA were coupled with changes in depressive symptoms during the study period. Future research should incorporate more comprehensive contextual covariates, extend follow-up beyond the first academic year, and use designs with more frequent assessments or intervention approaches to better examine within-person changes and causal mechanisms.

## Conclusions

This study examined depressive symptoms and intensity-specific leisure-time physical activity across the first academic year among first-year university students. Depressive symptoms were relatively elevated at university entry and showed a slight nonlinear decline over time, whereas overall LTPA increased across the academic year. At baseline, vigorous-intensity LTPA was significantly associated with lower levels of depressive symptoms, whereas the corresponding association for moderate-intensity LTPA was not statistically significant.

However, no significant change–change associations were observed between LTPA and depressive symptoms over time. These findings suggest that the relationship between LTPA and depressive symptoms in this sample was more evident at the level of baseline differences than in coupled change over time. In addition, gender, included as a time-invariant covariate, was associated with lower baseline LTPA levels among female students, but there was no evidence of gender differences in the longitudinal associations between LTPA and depressive symptoms.

Overall, the present findings support baseline associations rather than longitudinal or protective effects. Given the observational design and the possibility of unmeasured confounding, causal inferences cannot be drawn. Future research should incorporate more comprehensive contextual covariates, more frequent assessments, and stronger causal designs to better clarify the temporal relationship between changes in LTPA and changes in depressive symptoms during the transition to university.

## Supplemental Information

10.7717/peerj.21498/supp-1 Supplemental Information 1 Depression and Leisure-Time Physical Activity raw data All four measurement waves and the complete information from all scales.

10.7717/peerj.21498/supp-2 Supplemental Information 2 Codebook for raw data

10.7717/peerj.21498/supp-3 Supplemental Information 3 De-identified participant-level raw dataset including item-level questionnaire responses and variable coding for depressive symptoms and leisure-time physical activity (LTPA) across four waves among first-year students De-identified participant-level raw questionnaire responses collected across four waves among first-year university students. Variables include item- level measures of depressive symptoms and leisure-time physical activity (LTPA), including overall, vigorous-intensity, and moderate-intensity activity. A separate sheet in the file provides a codebook describing all variables, coding schemes, and scoring rules used in the study.

10.7717/peerj.21498/supp-4 Supplemental Information 4 Mplus input scripts (.inp) for latent growth and parallel-process latent growth models Mplus input files (.inp; UTF-8) used to reproduce the analyses in this study, including measurement invariance testing (PHQ-9), unconditional latent growth curve models (LGCM), and parallel-process latent growth models. Software required: Mplus (version 8.x or later). How to run: unzip the archive, place the corresponding .dat data file(s) in the same folder as each .inp file (or edit the path), then open the .inp file in Mplus and click Run. Data dependencies: the scripts reference the study dataset exported as .dat; variable order must match . Missing data code: 999. A README.txt file in the ZIP provides a file-by-file description and the referenced data filenames.

10.7717/peerj.21498/supp-5 Supplemental Information 5 STROBE checklist

## Additional Information and Declarations

### Data Availability

The following information was supplied regarding data availability: The raw measurements are available in the Supplemental Files.
