# Supplementary material for: Intensity-specific leisure-time physical activity and depressive symptoms among first-year university students: a four-wave longitudinal study
Source: PeerJ. 2026 Jul 2;14:e21498. doi: 10.7717/peerj.21498 (PMC13333126; doi:10.7717/peerj.21498)
Supplement: Supplemental Information 5 [file peerj-14-21498-s005.docx]

**STROBE Statement—Checklist of items that should be included in reports of cohort studies**

*Manuscript: Intensity-Specific Leisure-Time Physical Activity and Depressive Symptoms Among First-Year University Students: A Four-Wave Longitudinal Study*

|  | **Item No.** | **Recommendation** | **Location in manuscript** |
| --- | --- | --- | --- |
| **Title and abstract** | 1a | Indicate the study’s design with a commonly used term in the title or the abstract | Title, Page 1, Lines 1–2; Abstract/Methods, Page 2, Lines 29–33 |
|  | 1b | Provide in the abstract an informative and balanced summary of what was done and what was found | Abstract, Pages 2–3, Lines 23–48 |
| **Introduction** |  |  |  |
| Background/rationale | 2 | Explain the scientific background and rationale for the investigation being reported | Introduction, Pages 3–7, Lines 52–142 |
| Objectives | 3 | State specific objectives, including any prespecified hypotheses | Objectives, Pages 7–8, Lines 144–155 |
| **Methods** |  |  |  |
| Study design | 4 | Present key elements of study design early in the paper | Introduction, Page 5-7, Lines 92–143; |
| Setting | 5 | Describe the setting, locations, and relevant dates, including periods of recruitment, exposure, follow-up, and data collection | Methods/Participants and procedures, Pages 8–9, Lines 157–194 |
| Participants | 6a | Give the eligibility criteria, and the sources and methods of selection of participants. Describe methods of follow-up | Participants and procedures, Pages 8–9, Lines 158–194 |
|  | 6b | For matched studies, give matching criteria and number of exposed and unexposed | Not applicable; the study did not use a matched design |
| Variables | 7 | Clearly define all outcomes, exposures, predictors, potential confounders, and effect modifiers. Give diagnostic criteria, if applicable | Measures, Pages 10–12, Lines 203–240; Statistical analysis, Page 12, Lines 241–261 |
| Data sources/measurement | 8* | For each variable of interest, give sources of data and details of methods of assessment (measurement). Describe comparability of assessment methods if there is more than one group | Measures, Pages 10–12, Lines 201–240 |
| Bias | 9 | Describe any efforts to address potential sources of bias | Methods, Pages 9–10, Lines 191–201; Limitations, Page 30, Lines 621–646 |
| Study size | 10 | Explain how the study size was arrived at | Participants and procedures, Page 9, Lines 178–194 (available longitudinal cohort and analytic sample) |
| Quantitative variables | 11 | Explain how quantitative variables were handled in the analyses. If applicable, describe which groupings were chosen and why | Measures, Pages 10–12, Lines 203–240; Statistical analysis, Pages 12–13, Lines 241–261 |
| Statistical methods | 12a | Describe all statistical methods, including those used to control for confounding | Statistical analysis, Pages 12–13, Lines 241–261 |
|  | 12b | Describe any methods used to examine subgroups and interactions | Gender was included as a time-invariant covariate, Page 12, Lines 249–256, No formal subgroup or interaction analysis was conducted. |
|  | 12c | Explain how missing data were addressed | Missing data handling, Pages 9–10, Lines 191–201 |
|  | 12d | If applicable, explain how loss to follow-up was addressed | Page 9-10, Lines 178–201 |
|  | 12e | Describe any sensitivity analyses | Not applicable; no separate sensitivity analyses were conducted |
| **Results** |  |  |  |
| Participants | 13a | Report numbers of individuals at each stage of study—eg numbers potentially eligible, examined for eligibility, confirmed eligible, included in the study, completing follow-up, and analysed | Participants and procedures, Page 8-9, Lines 157–194 |
|  | 13b | Give reasons for non-participation at each stage | Participant retention/re-entry structure, Page 9, Lines 178–194 |
|  | 13c | Consider use of a flow diagram | No flow diagram included; participant retention is described in text on Page 9, Lines 178–194 |
| Descriptive data | 14a | Give characteristics of study participants (eg demographic, clinical, social) and information on exposures and potential confounders | Abstract/Methods, Page 2, Lines 29–30;Page 8, Lines 157–194, |
|  | 14b | Indicate number of participants with missing data for each variable of interest | Missing data and valid response counts, Pages 9–10, Lines 178–199 |
|  | 14c | Summarise follow-up time (eg, average and total amount) | Follow-up timing, Pages 8–9, Lines 170–177; total person-wave observations, Page 9, Lines 178–194 |
| Outcome data | 15* | Report numbers of outcome events or summary measures over time | Descriptive statistics and outcome summaries over time, Pages 13–14, Lines 262–293; growth model results, Pages 15–18, Lines 312–382 |
| Main results | 16a | Give unadjusted estimates and, if applicable, confounder-adjusted estimates and their precision (eg, 95% confidence interval). Make clear which confounders were adjusted for and why they were included | Main PP-LGM results, Pages 18–23, Lines 383–473 |
|  | 16b | Report category boundaries when continuous variables were categorized | PHQ-9 severity categories, Pages 10–11, Lines 203–220; LTPA intensity categories and scoring, Pages 11–12, Lines 221–240 |
|  | 16c | If relevant, consider translating estimates of relative risk into absolute risk for a meaningful time period | Not applicable; no relative risk estimates were reported |
| Other analyses | 17 | Report other analyses done—eg analyses of subgroups and interactions, and sensitivity analyses | No formal sensitivity analyses were conducted. |
| **Discussion** |  |  |  |
| Key results | 18 | Summarise key results with reference to study objectives | Discussion, Pages 23–29, Lines 475–619; Conclusions, Page 31, Lines 647–666 |
| Limitations | 19 | Discuss limitations of the study, taking into account sources of potential bias or imprecision. Discuss both direction and magnitude of any potential bias | Limitations, Pages 30–31, Lines 621–646 |
| Interpretation | 20 | Give a cautious overall interpretation of results considering objectives, limitations, multiplicity of analyses, results from similar studies, and other relevant evidence | Discussion, Pages 23–29, Lines 475–619; Limitations, Pages 30–31, Lines 621–646; Conclusions, Page 31, Lines 647–666 |
| Generalisability | 21 | Discuss the generalisability (external validity) of the study results | Limitations, Pages 30–31, Lines 621–646; Conclusions, Page 31, Lines 647–666 |
| **Other information** |  |  |  |
| Funding | 22 | Give the source of funding and the role of the funders for the present study and, if applicable, for the original study on which the present article is based | This study was supported by the 2026 Guangzhou Education Science Planning Project (Project No.  2026115144), titled “The Influencing Mechanisms and Guidance Strategies of College Students’ Sports  Participation Behavior from the Perspective of Online Social Communities.” |

* Give information separately for exposed and unexposed groups.

Note: The STROBE checklist is best used in conjunction with the STROBE Explanation and Elaboration article. Information on the STROBE Initiative is available at http://www.strobe-statement.org.
